# A New Non-Human Primate Model of Photochemically Induced Cerebral Infarction

**DOI:** 10.1371/journal.pone.0060037

**Published:** 2013-03-20

**Authors:** Satoshi Ikeda, Katsuhiro Harada, Akihiko Ohwatashi, Yurie Kamikawa, Akira Yoshida, Kazumi Kawahira

**Affiliations:** Department of Rehabilitation and Physical Medicine, Graduate School of Medical and Dental Sciences, Kagoshima University, Kagoshima, Japan; University of Queensland, Australia

## Abstract

**Background and Purpose:**

Rat models of photochemically induced cerebral infarction have been readily studied, but to date there are no reports of transcranial photochemically induced infarctions in the marmoset. In this report, we used this non-human primate as a model of cerebral thrombosis and observed the recovery process.

**Methods:**

Five common marmosets were used. Cerebral ischemia was produced via intravascular thrombosis induced by an intravenous injection of Rose Bengal and irradiation with green light. After inducing cerebral infarction, we observed the behavior of marmosets via a continuous video recording. We evaluated maximum speed, mean speed, and distance traveled in 1 min. In addition, we evaluated scores for feeding behavior, upper limb grip, and lower limb grip. We confirmed the infarct area after cerebral infarction using 2,3,5-triphenyltetrazolium chloride staining in a separate marmoset.

**Results:**

We found functional decreases 2 days after creating the cerebral infarction in all measurements. Total distance traveled, average speed, upper limb score, and feeding behavior score did not recover to pre-infarction levels within 28 days. Maximum speed in 1 min and lower limb score recovered 28 days after infarction as compared to pre-infarction levels. We confirmed the infarct area of 11.4 mm×6.8 mm as stained with 2,3,5-triphenyltetrazolium chloride.

**Conclusion:**

We were able to create a primate photothrombosis-induced cerebral infarction model using marmosets and observe functional recovery. We suggest that this is a useful model for basic research of cerebral infarction.

## Introduction

There are several ways to create cerebral infarction rat models. One such method is called photochemical induction, which reproducibly creates similarly sized infarcts in similar locations. This model also has a relatively low mortality rate. With respect to clinical rehabilitation, functional deficits resulting from stroke are long term. However, functional ability in the rat recovers to almost pre-infarct levels within 2 weeks after photochemical induction [1], [2]. A recent report that used a marmoset model of middle cerebral artery occlusion indicated that functional deficits were slow to recover [3]. To date, there are no reports of photochemically induced cerebral infarction in the marmoset.

In this study, we created a photochemically induced cerebral infarction marmoset model and observed the long-term effect of cerebral infarction. Working tasks are often used to evaluate motor behavior in cerebral infarction models, such as for the beam walking test in rats [1], [2] and the Hill and Valley Staircase test in marmosets [4], [5]. However, as these evaluation methods are affected by the repetition of the tasks, they are unsuitable for evaluation of the natural recovery process. In the current study, we evaluated the recovery process using continuous video recordings in order to eliminate the effect of task repeating.

The adult marmoset has a brain approximately 4 times the size of the rat with same body weighs of 250 to 400 g. Marmosets are readily bred and are generally easy to handle, which is advantageous for behavioral testing and postoperative management [6]. In addition, Sasaki et al. were successful in creating a transgenic marmoset [7]. Keeping these points in mind, we concluded that a stroke model in the marmoset would be useful for cerebral infarct research. In previous studies, marmoset models were created through direct cerebral injury by opening the skull, as well as by internal carotid artery occlusion. In these studies, ipsilateral and contralateral disorders were observed in the marmosets during the acute phase 1 week after the onset of cerebral infarction. These models showed disabilities even in the sub-acute phase 45 days from cerebral infarction. The cerebral infarction model induced by middle cerebral artery occlusion is useful for many studies; however, permanent infarction models have high mortality, and about 30% of rats in such models die [8]–[10]. Infarct volume size varies among individuals induced by middle cerebral artery occlusion because of the variability in collateral blood flow. Photochemical induction of cerebral infarction, on the other hand, is relatively less invasive and has a low mortality rate. It is also easy to set up the infarct area within an objective range. For these reasons, photochemically induced rat and mouse models are frequently used to evaluate the effects of drugs and rehabilitation [11]. Considering that this model has a small variation in infarct size and a low mortality rate when compared with previous study models, we created a photochemically induced cerebral infarction model of the non-human primate marmoset and evaluated its behavior using video recordings.

## Materials and Methods

All experimental procedures were conducted in accordance with the ethical guidelines for animal experimentation of Kagoshima University, the National Institutes of Health, and the use of non-human primates in research. This study was approved by the animal experiment committee of Kagoshima University (approval number: MD11057).

Animals were housed in cages in an environmentally controlled room with a 12/12 h light/dark cycle and received disinfectant treatment once a day. Animals were given adequate amounts of food and water until they recovered their ability to ingest food and water without assistance; thereafter, they had free access to food and water in the cage.

We used 5 marmosets for behavior observation and 1 additional marmoset to confirm the infarct areas. Before inducing infarction, we determined the dominant hand of each animal from its feeding behavior and induced infarction in the contralateral hemisphere in order to achieve hemiparesis of the dominant hand.

### Creating Infarction

We created the infarction in the marmosets’ left hemispheres since all animals primarily used the right forelimb in feeding behavior. Cerebral ischemia was produced via intravascular thrombosis induced by an intravenous injection of Rose Bengal (20 mg/kg) and irradiation with green light (533 nm, metal halide lamp, PCS-UMX350; Nippon P.I Co., Ltd., Tokyo, Japan) for 5 min under deep anesthesia. All surgeries were performed under general anesthesia induced by an intramuscular injection of ketamine (50 mg/kg; Sankyo, Tokyo, Japan) and maintained by isoflurane (Foren; Abbott, Tokyo, Japan). To target the sensorimotor area of the cerebral cortex, green light of 8 mm diameter was irradiated on the exposed skull at 6 mm lateral to the midline and a midpoint of the anteroposterior diameter of the skull. Rose Bengal was injected via the tail vein. Platelet aggregation induced by Rose Bengal and green light has been reported to take only 2 min [12]. To avoid an increase in radiation field temperature, we irradiated for 5 min. We kept the radiation field within 38 degrees using a monitoring thermometer, dripping saline, and a fan blower.

### Determining Infarct Volume

We stained the brain of 1 marmoset with 2,3,5-triphenyltetrazolium chloride (TTC) to measure the infarction volume. After ketamine induction, the animal was given an overdose of pentobarbital. The marmoset’s brain was then removed and sliced into 3-mm-thick coronal sections. These sections were immersed in a 2% solution of TTC at 37°C for 20 min to reveal the infarcted area. After that, we photographed the surface of the brain and each cross-section. We measured the diameters of the major and minor axes as well as the depth and area of the infarction on the cerebral surface using image analysis software (Image J, U. S. National Institutes of Health, Bethesda, Maryland, USA) and calculated the infarct volume using an ellipsoid formula.

At 28 days after infarction, animals were perfused transcardially with 300 ml of 4% paraformaldehyde in 0.1 M phosphate buffer at pH 7.4 under deep anesthesia. The brains were removed and post-fixed in perfusion fixative overnight. Nissl staining was then performed done and infarction volume was measured.

### Evaluation

Recently, Erickson et al. observed rats by video and evaluated them via frame-by-frame analysis [13]. In the current study, we evaluated behavioral scores and behavior analysis by recording video data, in order to eliminate the learning effect of repeating the working tasks.

### Video Recording

The breeding cage was made of wire mesh, and the marmosets could move freely and catch the mesh. The cage had a wooden board and a wooden rod, with a bait box in front of the wooden rod. Several days after surgery, a dish was prepared with milk. Marmosets were recorded using webcams (Webcam c200; Logicool Co., Ltd) with Windows personal computer and recording software (Windows Movie Maker version 5.1, Microsoft R) at 15 frames/s continuously for 24 h. We repeatedly recorded for 28 days.

### Behavioral Analysis

We analyzed the behavior of the marmosets, including feeding motion, with analysis software (DIPP-MOTION-PRO; Ditect Co.) for 1 min. Maximum speed, mean speed, and distance traveled were calculated.

We also evaluated feeding behavior with the following score scheme: Score 4: use of the contralateral forelimb; Score 3: use of the ipsilateral forelimb; Score 2: ate the pellet directly without using forelimb; Score 1: licked the milk of the dish directly; and Score 0: unable to eat. The forelimb grip scores were designated as follows: Score 2: able to catch the wire mesh of cage without slipping; Score 1: able to catch the wire mesh of cage but with a slipping grasp; and Score 0: unable to catch the wire mesh of cage. The hind limb grip scores were designated as follows: Score 2: able to ride the wooden perch without foot drop; Score 1: foot drop but able to ride the wooden perch; Score 0: unable to ride the wooden perch. These scores were modified using the neurologic scoring of Marshall and Ridley [5]. The total behavior score was the sum of these scores; the highest score possible was 8 and the lowest score 0. These evaluations were done with recorded video images. Similar photochemical induced cerebral infarction models have been previously established in other animals. Considering animal welfare, we did not make sham-operated animals. At the end of this study, the marmosets were injected with lethal dose of pentobarbital after Ketamine induction to be sacrified.

Statistical analyses were done using analysis of variance (ANOVA) and Fisher’s protected least significant difference test.

## Results

**Figure 1:**
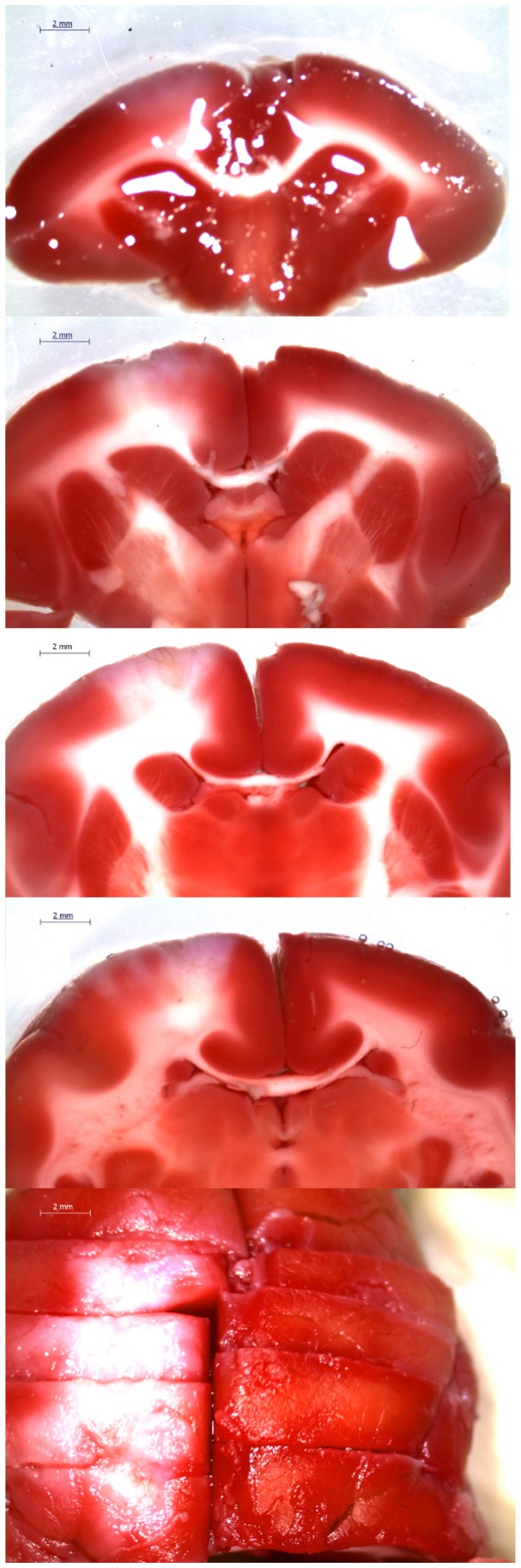
TTC (2,3,5-Triphenyltetrazolium chloride) staining. Normal region of mitochondrial activity was stained, while the ischemic area was not.

We confirmed the infarct area with TTC staining. At 24 h after surgery, we measured the surface and cross-sectional area of the cerebral infarction. The length diameter was 11.4 mm and the width diameter was 6.8 mm. Surface infarction area was 66.9 mm^2^. The cross-sectional image revealed that the infarct was 2.6 mm in depth with a cross-sectional area of 9 mm^2^. The infarct volume was calculated to 105.5 mm^3^ using the equation of the ellipsoid volume (Fig. 1).

**Figure 2:**
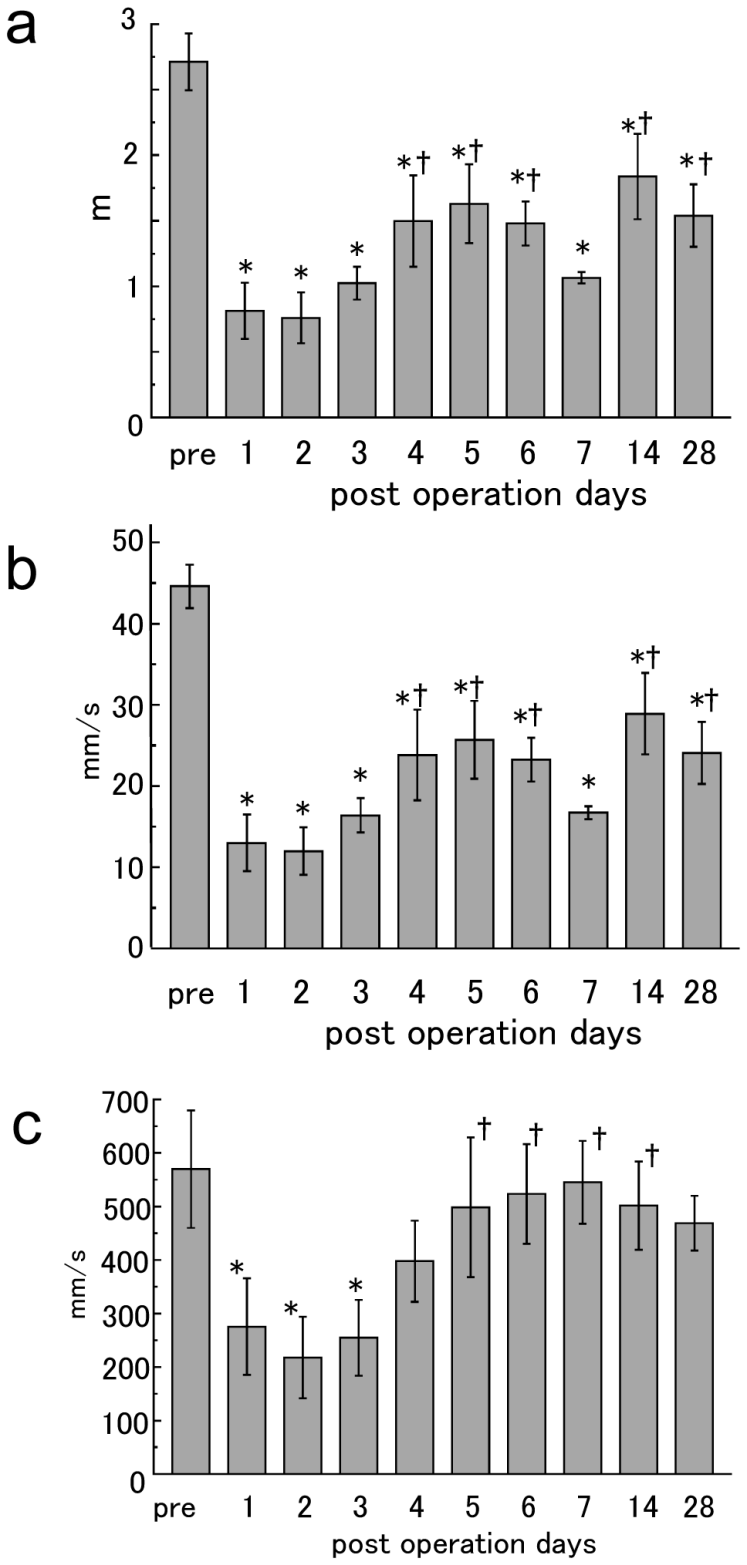
Behavioral analysis including feeding motion. a) Distance traveled per minute. We show the mean value and standard error of total distance traveled, including feeding behavior, for a 1-min period 28 days after surgery. b) Mean speed/min. The mean and standard error of the moving speed during 1 min 28 days after operation. c) Maximum speed/min. The mean and standard error of the maximum speed, including feeding behavior, for 1 min 28 days after operation. * P<0.05 in comparison with preoperative values indicating presence of deficit. † P<0.05 in comparison with the lowest value (2 days after operation) indicating recovery.

**Figure 3:**
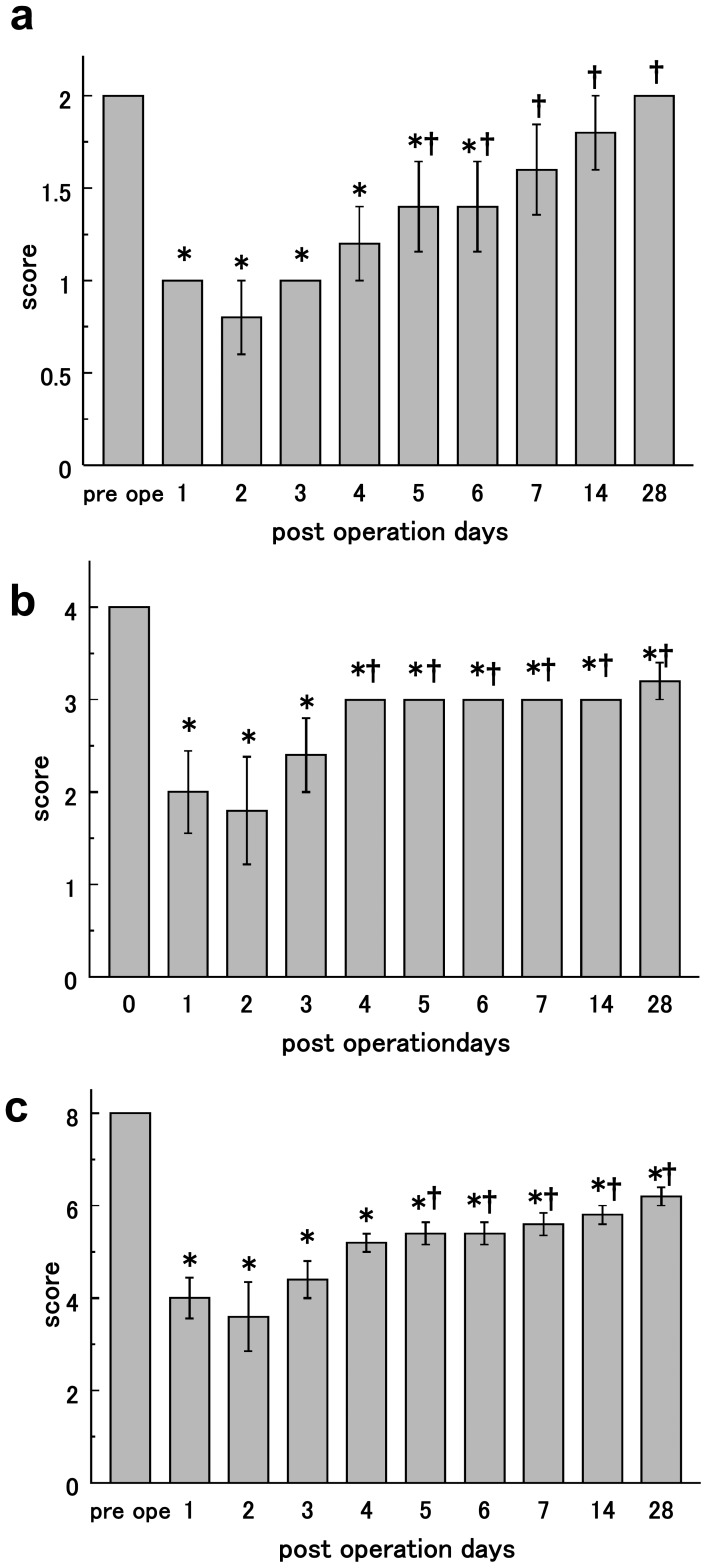
Behavior score. a) Hind limb score. The mean and standard error of lower limb scores pre-surgery to postoperative day 28. b) Feeding behavior score. The mean and standard error of feeding behavior scores pre-surgery to postoperative day 28. c) Total behavior score. The mean and standard error of the total score of feeding behavior, upper limb grip, and lower limb. * P<0.05 in comparison with preoperative values indicating presence of deficit. † P<0.05 in comparison with the lowest value (2 days after operation) indicating recovery.

Before the operation, the marmosets moved 2.71±0.21 m/min; this value was reduced to 0.76±0.19 m/min 2 days after operation. The value naturally recovered to an average of 1.53±0.23 m/min after 28 days. Total distance traveled significantly decreased 1 day after creating the infarction, but began recovering 3 days later. However, this value was still lower than pre-infarction levels 28 days after the operation, ANOVA value : F = 6.03, P<0.0001,Sample size n = 5, Degrees of freedom 9, Sum of the squares 1.47×10^7^, Mean square 1.66×10^6^ (Fig. 2a). The mean moving speed for 1 min was 44.6±2.68 mm/s in normal marmosets. Most animals had reduced speed on the second day after surgery at 11.9±2.92 mm/s; 28 days later, it was 24.0±2.82 mm/s. The speed significantly decreased from the first day after infarction induction, and it continued to decrease until day 28, ANOVA value : F = 6.74, P<0.0001,Sample size n = 5, Degrees of freedom 9, Sum of the squares 4.07×10^3^, Mean square 4.52×10^2^ (Fig. 2b). The maximum speed in 1 min, prior to the infarction, was 570±110 mm/s. On day 2 after surgery, it was reduced to 218±76.3 mm/s. After 28 days, it improved to 469±50.8 mm/s and was not significantly different from the pre-infarction value, ANOVA value : F = 2.19, P = 0.044,Sample size n = 5, Degrees of freedom 9, Sum of the squares 7.67×10^5^, Mean square 8.52×10^4^ (Fig. 2c). Regarding the evaluation of the forelimb grip score, the right forelimb did not slip the wire mesh of the cage before the infarction in all 5 animals. After infarction induction, grip failures were evident on the day after infarction and sustained until 28 days after operation. During the preoperative session, marmosets could balance on their hind limbs on the rod without slipping. Marmosets’ hind limb functions worsened on day 2 after infarction, and they could not ride the wooden perch. After 28 days, the marmosets had recovered enough to be able to place their hind limbs on a wooden perch without slipping, ANOVA value: F = 6.06, P<0.0001,Sample size n = 5, Degrees of freedom 9, Sum of the squares 8.18, Mean square 0.91 (Fig. 3a).

We also evaluated the feeding behavior of the marmosets. Two days after inducing the cerebral infarction, the functional feeding behavior score was its lowest; the marmosets ingested milk from the top of the dish directly. After the fourth day, the marmosets had recovered enough to eat using the left forelimb. After 28 days, they still did not use the right forelimb in feeding behavior, ANOVA value : F = 5.38, P<0.0001,Sample size n = 5, Degrees of freedom 9, Sum of the squares 17.9, Mean square 1.99 (Fig. 3b). The total score was significantly reduced from the pre-infarction value through postoperative day 2. Then, the total score improved significantly through day 28. However, the total score did not recover to the same level as before surgery, ANOVA value: F = 12.6, P<0.0001,Sample size n = 5, Degrees of freedom 9, Sum of the squares 69.1, Mean square 7.68 (Fig. 3c).

**Figure 4:**
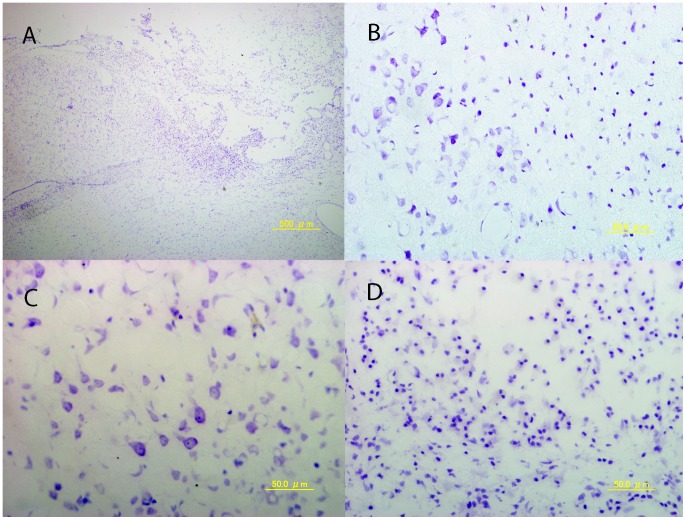
Photomicrograph of cerebral cortex 28 days after photochemical infarction with Nissl staining. a) Low power field; b) border area of infarction; c) intact cortex; and d) infarcted area with high power field.

After fixation, an infarction scar was seen in frontal lobe. The average length diameter was 7.43±0.28 mm and the average width diameter was 4.26±0.84 mm. Surface infarction area was 27.9±4.03 mm^2^. The cross-sectional image showed that the infarct was 2.15±0.12 mm in depth with a cross-sectional area of 6.07±1.10 mm^2^. The infarct volume was calculated to 38.00±6.27 mm^3^ using the equation of the ellipsoid (Fig. 4).

## Discussion

We previously evaluated recovery from hemiplegia and expression of brain-derived neurotrophic factor (BDNF) using a photochemically induced cerebral infarction model of the rat [2]. There are several advantages of using the photochemically induced cerebral infarction model. Cerebral infarction can be created without opening the skull, thus lowering risk of intracranial infection. Moreover, infarcts of similar size and in similar locations can be created across individual animals. This model also has a high survival rate due to lower surgical stress.

In the current study, we made 2 modifications to create photochemically induced cerebral infarctions in the marmoset. First, because marmosets’ skulls are thicker than rats, it was difficult for green light to reach the deep tissue. Therefore, we changed the light output intensity from 150 W (the intensity used on rats) to 350 W. Second, to avoid an excessive rise in temperature rise to the high intensity light, we minimized the irradiation time and used water and blower cooling methods.

In the rat infarction model, animals recovered in about 2 weeks [1]. However, hemiplegia after cerebral infarction often persists for more than 6 months in severe human cases [14]. In this study, we used the common marmoset, which is closer to the human species than the rat. Hemiplegia developed after cerebral infarction, and we evaluated the recovery process of the marmosets. Feeding behavior was reduced to a level where the marmosets could only lick milk directly from the dish on the second day after infarction. Then, they recovered to the level that they could take and eat pellets using the left unaffected forelimb 28 days after infarction. However, animals did not use the right hemiparetic forelimb. Our results are in agreement with previous work by Freret et al. [4], who reported that in a model of cerebral infarction created by middle cerebral artery occlusion, marmosets’ function of both forelimbs was reduced immediately after surgery, and the function of the unaffected side forelimb recovered after 3–4 days. In the current study, marmosets were alive and observed for 28 days, and in this time we were able to observe the marmosets’ activity; forelimb and hind limb function on the paralyzed side declined. Compared to observations in rat models, these results are closer to the clinical course of humans. Our results suggest that this model can be used to observe the effects of medication and rehabilitation.

Improvement in hind limb function was observed, and maximum speed and hind limb scores 28 days after infarction were not significantly different from the pre-infarction values. On the other hand, feeding behavior was restricted to the left side (non-hemiplegia side) 28 days after cerebral infarction. In the forelimb functional evaluation, we observed the right forelimb (hemiplegia side) slipping from the wire mesh. From these findings, we concluded that deficits of the forelimb function in this model were sustained after 28 days. These results suggest that on functional localization, the area responsible for forelimb function sustained more damage than the area responsible for hind limb function. In addition, maximum movement speed recovered to pre-infarction levels within 4 weeks, whereas distance traveled and average speed was not restored to pre-infarction levels. We hypothesize that these results stem from the reduction in overall activity.

Although there have been many reports on the correlation of functional decline and infarct area, it has been difficult to calculate correlations because of the various factors that affect functional recovery, such as size of the infarct area, natural recovery, functional localization, and learning effects in behavioral tasks. For this reason, it has been difficult to clearly demonstrate the correlation of functional decline and infarct area [15]. In the observational behavioral assessment of marmosets after cerebral infarction used in the current study, functional improvement measurements that were confounded by learning effects due to repeated tests were eliminated.

Bihel et al., using a model of temporary occlusion recanalization after middle cerebral artery occlusion, illustrated the correlation between magnetic resonance images and functional recovery in the chronic phase using fiber tracking and angle projection in the slice plan of the first eigenvector [3]. Ramos-Cabrer et al. examined the effect of stem cell transplantation to cortical infarcted areas in rats that survived gray matter infarction induced by middle cerebral artery occlusion [10]. However, it is bit difficult to sufficiently damage the grey matter as well as obtain survivors using middle cerebral artery occlusion. The photochemically induced cerebral infarction model is exceptionally useful as it can be used to damage gray matter to a greater extent than white matter and is associated with an excellent survival rate.

There are many studies using cerebral infarction rodent models. Neurotrophic factors and the introduction of stem cells were recently reported as being involved in neonatal and regeneration of nerve cells in the brain [10], [16]. Bejot et al. reported that, in brain infarctions, BDNF concentrations in the blood and brain were not correlated, and BDNF concentration in rats increased on severe cerebral infarction [17]. Furthermore, these authors reported the activity of non-neuronal BDNF-producing cells after cerebral infarction in rat models [18]. Therapeutic interventions for cerebral infarction also include therapeutic hypothermia. Clinically, the correlation of body temperature and prognosis has been pointed out. However, hypothermia has not been established as a therapeutic method [19]. Rats or other rodents are typically used in basic hypothermia research [20], but these results may not accurately predict human responses. In this respect, since the infarction model in the marmoset is more closely related to human disease, we conclude that the photochemically induced cerebral infarction model in primates is useful to promote these studies.

### Conclusions

We established a primate model of cerebral infarction with photochemical induction. With this model, we obtained a near-homogeneous lesion and similarly protracted functional deficiency as seen in humans. This model is practical for studying pharmacological and rehabilitative effects, as well as various kinds of brain functions following cerebral infarction.
